# PKM2 Is Required to Activate Myeloid Dendritic Cells from Patients with Severe Aplastic Anemia

**DOI:** 10.1155/2018/1364165

**Published:** 2018-02-15

**Authors:** Chunyan Liu, Mengying Zheng, Ting Wang, Huijuan Jiang, Rong Fu, Huaquan Wang, Kai Ding, Qiufan Zhou, Zonghong Shao

**Affiliations:** ^1^The Department of Hematology, General Hospital of Tianjin Medical University, Tianjin, China; ^2^The Department of Hematology, The Second Hospital of Tianjin Medical University, Tianjin, China

## Abstract

Severe aplastic anemia (SAA) is an autoimmune disease in which bone marrow failure is mediated by activated myeloid dendritic cells (mDCs) and T lymphocytes. Recent research has identified a strong immunomodulatory effect of pyruvate kinase M2 (PKM2) on dendritic cells in immune-mediated diseases. In this study, we aimed to explore the role of PKM2 in the activation of mDCs in SAA. We observed conspicuously higher levels of PKM2 in mDCs from SAA patients compared to normal controls at both the gene and protein levels. Concurrently, we unexpectedly discovered that after the mDC-specific downregulation of PKM2, mDCs from patients with SAA exhibited weakened phagocytic activity and significantly decreased and shortened dendrites relative to their counterparts from normal controls. The expression levels of the costimulatory molecules CD86 and CD80 were also reduced on mDCs. Our results also suggested that PKM2 knockdown in mDCs reduced the abilities of these cells to promote the activation of CD8+ T cells (CTLs), leading to the decreased secretion of cytotoxic factors by the latter cell type. These findings demonstrate that mDC activation requires an elevated intrinsic PKM2 level and that PKM2 improves the immune status of patients with SAA by enhancing the functions of mDCs and, consequently, CTLs.

## 1. Introduction

Severe aplastic anemia (SAA) is a hematologic disease characterized by pancytopenia with severe bone marrow failure. To date, an increasing number of studies have recognized SAA as an autoimmune disease in which bone marrow failure is mediated by activated T lymphocytes [1, 2]. Myeloid dendritic cells (mDCs) have recently been recognized as important players in the primary immune responses related to SAA. Our previous research demonstrated increases in both the immature and activated mDC populations in the bone marrow of SAA patients, indicating that immune imbalances might originate from an early stage in the antigen recognition process [3]. Stimulated mDCs secrete IL-12 and thus act as major stimulators of the polarization of Th0 cells to Th1 cells, a process that leads to excessive T lymphocyte function and ultimately to the apoptosis of hematopoietic cells. Although knowledge about the immunopathogenesis of SAA has improved gradually after years of research, the specific mechanism by which activated mDCs and even T cells are involved requires further validation. Consequently, the immune etiology of SAA has become the focus of further research.

Within the glycolytic pathway, pyruvate kinase M2 (PKM2) catalyzes the dephosphorylation of phosphoenolpyruvate to pyruvate, a rate-limiting step [4, 5]. PKM2 therefore acts as a key regulator of metabolic activities in both cancer and activated immune cells, with critical roles in cell growth, proliferation, apoptosis, and many other physiological activities [6, 7]. PKM2 can be allosterically regulated by metabolites and intracellular signaling pathways, and previous observations have indicated that PKM2 may interact with some pathogen-related proteins at the chromatin level (e.g., staphylococcal Opa, human immunodeficiency virus, and hepatitis C virus) to enhance their pathogenicity and subsequently promote disease progression [8–10]. Additionally, recent research has shown that PKM2 has a strongly immunomodulatory effect on the antigen-presenting abilities of dendritic cells [11]. However, the relationship between PKM2 and mDCs in the context of SAA remains unclear. In this study, we aimed to investigate the role of PKM2 in mDC activation in SAA patients and to provide data to support a potential mechanism of mDC activation and the immune process in this population.

## 2. Materials and Methods

### 2.1. Study Subjects

Thirty patients with SAA, including 12 males and 18 females with a median age of 37 years (range, 10–58 years), were enrolled in the present study. All patients, including 15 newly diagnosed cases and 15 cases in remission after immunosuppressive therapy (IST), had been diagnosed according to International AA Study Group criteria at the Department of Hematology, Tianjin Medical University General Hospital, Tianjin, between September 2014 and November 2015. The disease was considered severe (i.e., SAA) if at least two of the following parameters were met: a neutrophil count < 0.5 × 10^9^/L, platelet count < 20 × 10^9^/L, and reticulocyte count < 20 × 10^9^/L with hypocellular bone marrow. Cases with a neutrophil count < 0.2 × 10^9^/L were diagnosed as very SAA (VSAA). Patients were excluded if they had congenital AA or other autoimmune diseases. All patients were screened for paroxysmal nocturnal hemoglobinuria (PNH) by flow cytometry with anti-CD55 and anti-CD59 antibodies, and no PHN clones were identified. Remission was defined as improvement of AA after treatment with immunosuppressive therapies (e.g., anti-thymocyte globulin, cyclosporine, and glucocorticoid) and hematopoietic-stimulating factors (e.g., granulocyte colony-stimulating factor, recombinant human erythropoietin, recombinant human thrombopoietin, and/or IL-11). All patients in remission achieved a bone marrow hematopoietic recovery and became transfusion-independent, although some patients with normal peripheral blood cell counts continued to require drug therapy.

Eighteen healthy volunteers (10 males, 8 females) with a median age of 26 years (range, 23–40 years) were selected as normal controls. This study was approved by the Ethics Committee of Tianjin Medical University. Informed written consent was obtained from all patients in accordance with the Declaration of Helsinki.

### 2.2. Cell Culture and Purification

The targeted bone marrow mononuclear cells (BMMNCs) were extracted from SAA patients and healthy volunteers by density gradient centrifugation using a Ficoll-Paque PLUS solution (Amersham Biosciences, Uppsala, Sweden). Cells from each subject were cultured separately at a density of 2 × 10^6^ cells/mL in complete medium [RPMI 1640 culture medium supplemented with 10% fetal bovine serum (FBS, Hyclone, Logan, UT, USA), 60 mg/L penicillin and 100 mg/L streptomycin (Gibco BRL, Grand Island, NY, USA)] containing 100 ng/mL recombinant human (rh) granulocyte-monocyte colony-stimulating factor (GM-CSF) and 40 ng/mL rhIL-4 (PeproTech Inc., Rocky Hill, NJ, USA) at 37°C in an atmosphere containing 5% CO_2_. Fresh medium and cytokines were added every 2 days. On day 6, rh tumor necrosis factor (TNF) (1000 *μ*g/mL) (PeproTech Inc.) was added for 24 h to induce mDC maturation. On day 7, suspended mature mDCs were collected from the culture supernatant and stained with FITC-conjugated human leukocyte antigen- (HLA-) DR FITC and APC-conjugated CD11c-APC-specific monoclonal antibodies. HLA-DR + CD11c+ cells were sorted and collected using a FACSAria flow cytometer (BD Biosciences, San Jose, CA, USA), and the purity of the sorted cells was determined.

### 2.3. RNA Isolation and qPCR

Total RNA was extracted using TRIzol reagent (Invitrogen, Carlsbad, CA, USA) according to the manufacturer's protocol. Equal amounts of RNA were reverse transcribed using the iScript cDNA Synthesis kit (Bio-Rad, Hercules, CA, USA). Real-time PCR was performed using 1 *μ*L of each cDNA working solution in a final volume of 25 *μ*L containing 12.5 *μ*L of SYBR solution (Invitrogen) and sense and antisense primers (300 nM each). *β*-Actin was used as a housekeeping gene with which to standardize the expression levels of targeted mRNAs. PCRs were performed on a Bio-Rad PCR iQ5 device (Bio-Rad), using the following thermal cycling profile for all genes of interest: 95°C for 2 min, followed by 40 amplification cycles (95°C for 10 s, the indicated annealing temperature for 35 s, 72°C for 30 s, and 65°C for 10 s). The primer sequences and annealing temperatures are listed in Table 1. The relative expression levels of all genes of interest were calculated using the 2^−ΔΔCt^ method.

### 2.4. Western Blotting

Cultured mDC cells were collected and lysed directly in RIPA buffer supplemented with a complete protease inhibitor (Roche, Basel, Switzerland) and phosphatase inhibitors (Roche). Protein levels in the lysates were quantified using a BCA kit. Proteins were separated via 10% sodium dodecyl sulfate polyacrylamide gel electrophoresis (SDS-PAGE) and transferred to nitrocellulose (NC) membranes (Millipore Corp., Billerica, MA, USA). The membranes were blocked with 10% skimmed milk (BD Biosciences) and subsequently incubated with anti-PKM2 (R&D Systems, Minneapolis, MN, USA) and anti-*β*-actin antibodies (Cell Signaling Technology, Danvers, MA, USA) at a dilution of 1 : 2000. The antibodies were dissolved in a solution containing 5% dried milk in Tris-buffered saline with Tween 20 (TBS-T) (20 mmol/L Tris–HCl buffer, pH 7.4, 150 mmol/L NaCl, 0.05% Tween 20). After extensive washing with phosphate-buffered saline (PBS), the membranes were then incubated with relevant horseradish peroxidase-conjugated secondary antibodies (1 : 1000 dilution; Zhongshan Biotech, Beijing, China). The labeled protein bands were detected using Super ECL Plus Detection Reagent. All protein levels were normalized to *β*-actin.

### 2.5. siRNA Transfection

Once purified, mDC cells from SAA patients were transfected with control (siControl) or PKM2 siRNA via Lipofectamine (Invitrogen) for 72 h. The siRNA oligos were synthesized by Sigma (St. Louis MO, USA). The transfected cells were collected and functionally evaluated by western blotting. The ultrastructures on mDCs were observed using a scanning electron microscope (SEM).

### 2.6. Phagocytosis

To study phagocytosis, mDCs were incubated in the dark with carboxylate-modified yellow-green (YG) microspheres (9 × 10^6^ beads; diameter, 2 *μ*m) for 1.5 hours at 37°C. After washing with PBS, the cells were analyzed on a FACSCalibur flow cytometer (BD Biosciences) with excitation/emission wavelength settings of 488/530 nm to count the microsphere-containing mDCs by detecting the wavelength emissions of the fluorescent microspheres. The percentage of phagocytosis (PP) was determined using the following formula: PP = M1 + M2 + M3 + M4, where M1, M2, M3, and M4 corresponded to the numbers of cells with one, two, three, and four microspheres, respectively. The phagocytosis index (PI) was determined as follows: (intracellular cpms/(intracellular + extracellular cpms)) × 100%.

### 2.7. Detection of CD80 and CD86 Molecular Expression by Flow Cytometry

Surface expression of the costimulatory molecules CD80 and CD86 on mDC cells was evaluated by flow cytometry to verify the effect of PKM2 siRNA transfection on the activation and function of mDCs from patients with SAA. HLA-DR-FITC, CD11c-APC, CD86-PE, and CD80-PE antibodies and appropriate isotypic controls (BD PharMingen, San Diego, CA, USA) were added to the sample tubes according to the manufacturer's instructions. All sample data were acquired using a FACSCalibur flow cytometer and analyzed using CellQuest software, version 3.1 (BD Biosciences).

### 2.8. Cell Coculture Assays

CD8+ T cells (i.e., CTLs) from SAA patients were sorted by immunomagnetic separation. To verify the effects of PKM2 on the ability of mDCs from patients with SAA to activate CD8+ T cells, mDCs treated with PKM2-siRNA or siControl were cocultured with CTLs at a ratio of 1 : 4 for 72 hours. All culture supernatants were collected, and the IFN-*γ* levels were quantified using commercial enzyme-linked immunosorbent assay (ELISA) kits according to the manufacturer's instructions (R&D Systems). Additionally, the expression of *perforin* and *granzyme B* mRNA in cocultured CTL cells was analyzed by quantitative RT-PCR as described above. The primers used to amplify *perforin*, *granzyme B*, and *β-actin* are listed in Table 2. An apoptosis analysis of cocultured CTLs was performed using a FITC Annexin V apoptosis detection kit I (BD Biosciences, Franklin Lakes, NJ, USA), followed by analysis on a FACSCalibur flow cytometer.

### 2.9. Statistical Analysis

Data are presented as means ± standard deviations. The significance of differences between groups was assessed using Student's *t*-test. A *P* value of <0.05 was considered statistically significant. All statistical analyses were performed using the SPSS 21.0 statistical package (SPSS Inc., Chicago, IL, USA).

## 3. Results

### 3.1. Elevated PKM2 mRNA and Protein Expression in mDC Cells from SAA Patients

**Figure 1:**
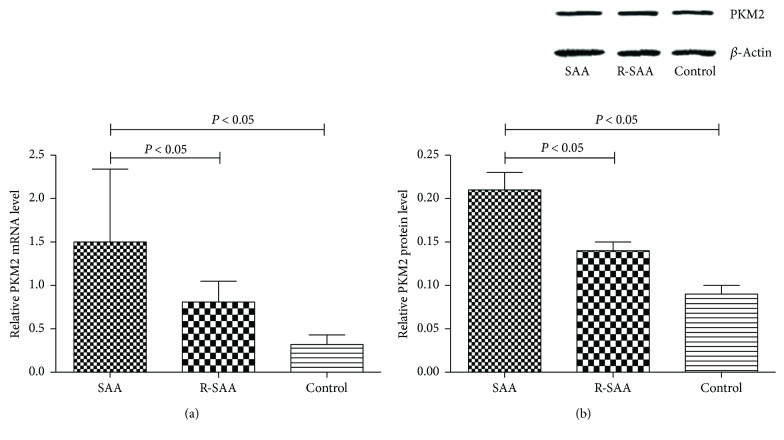
The expression level of PKM2 mRNA and protein by qPCR (a) and western blot (b). Proteins were detected with monoclonal anti-polyhistidine antibodies. We found increased PKM2 expression in untreated SAA patients.

Both western blotting and qPCR were used to evaluate the expression of PKM2 mRNA and protein in mDC cells from untreated SAA patients, patients in remission, and normal controls. As shown in Figure 1(a), significantly higher expression of *PKM2* mRNA was observed in mDC cells from untreated SAA patients (1.50 ± 0.84), compared to cells from patients in remission (0.81 ± 0.24) and controls (0.32 ± 0.11, *P* < 0.05). Western blotting revealed a similar trend in protein expression (Figure 1(b)).

### 3.2. Downregulation of PKM2 Strongly Impairs mDC Function

To verify whether mDC-specific PKM2 overexpression was responsible for the activation of mDCs in SAA, we successfully silenced PKM2 gene expression in this cell population via siRNA transfection. This process resulted in significantly lower levels of PKM2 protein expression relative to cells transfected with siControl.

**Figure 2:**
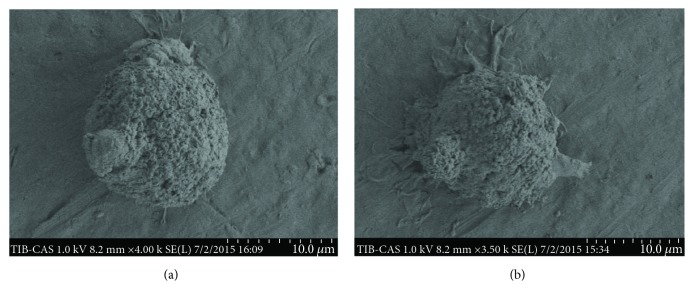
mDCs were visualized identified by SEM: (a) the shape of typical round mDCs after PKM2 siRNA transfection (original magnification: 5000x); (b) a more matured mDC in the siRNA-control group (original magnification: 5000x).

We used a SEM to evaluate differences in the ultrastructures on mDCs from the PKM2-siRNA and siControl groups. Following PKM2-siRNA transfection, the mDCs were round-shaped, with a typical morphology of small, short protrusions. Conversely, the mDCs from the siControl group had long, branched protrusions (Figure 2).

**Figure 3:**
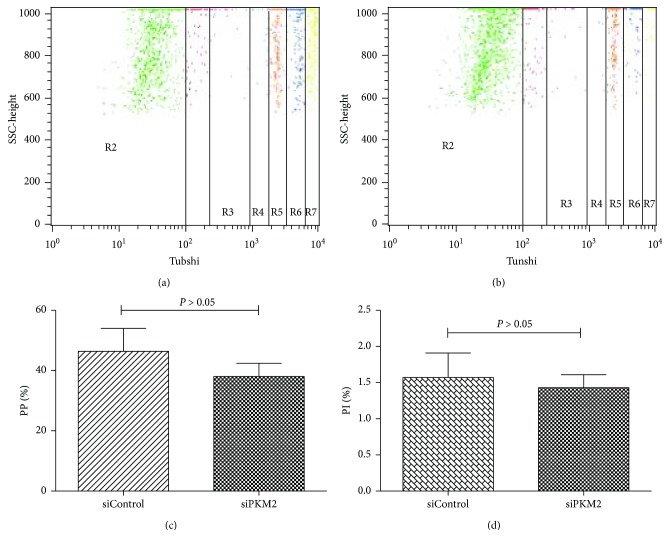
FACS detection on antigen uptake capacity of mDCs. (a) Antigen uptake capacity of mDCs in the siControl group. (b) Antigen uptake capacity of mDCs in the siPKM2 group. (c) The level of PP of mDCs from the siControl group was higher than that of the siPKM2 group (*P* < 0.05). (d) There was no statistical difference of PI between the two groups (*P* > 0.05).

Antigen uptake and processing is a typical hallmark of mDCs. Notably, we observed that mDCs transfected with PKM2 siRNA had a lower phagocytic ability (Figure 3). The PP and PI of mDCs in the siControl group were 46.37% ± 7.6% and 1.57 ± 0.34, respectively, whereas the corresponding levels in mDCs transfected with PKM2 siRNA were 38.02% ± 4.4% and 1.43 ± 0.18, respectively. This difference in PP was significant (*P* < 0.05). Although the intergroup difference in PI was not significant, we observed a decreasing trend after PKM2 siRNA transfection (*P* > 0.05). These results suggest that PKM2 downregulation decreased the antigen uptake capacities of mDCs.

**Figure 4:**
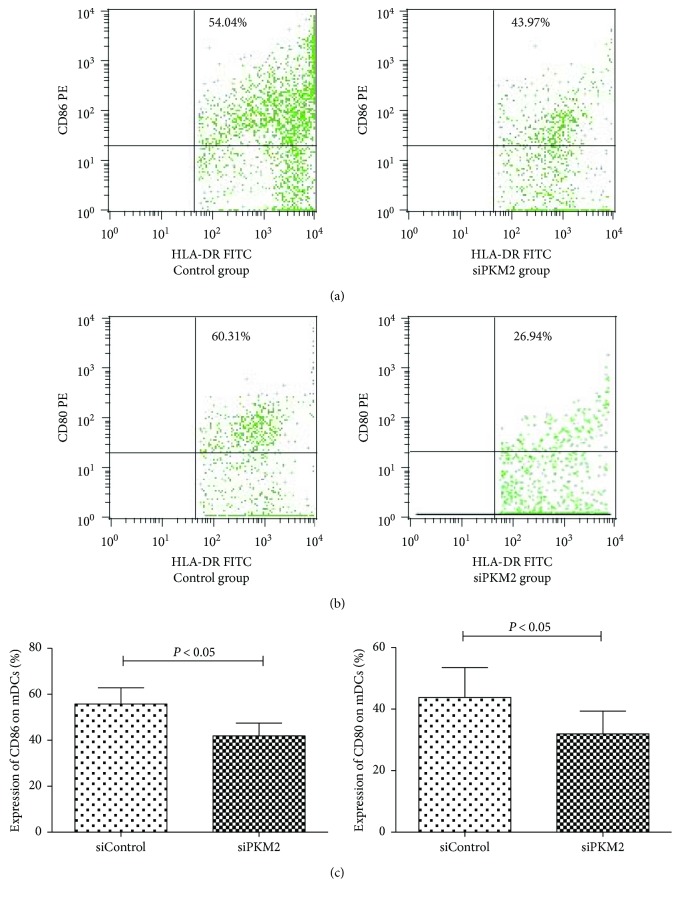
The levels of costimulatory molecules CD80 and CD86 on mDCs in the siControl group and siPKM2 group by flow cytometry. The levels of CD86 and CD80 on mDCs from the siControl group were higher than those from the siPKM2 group (*P* < 0.05).

Next, we functionally examined the expression of the costimulatory molecules CD80 and CD86 on the surfaces of mDCs by flow cytometry. We observed a significantly lower frequency of CD86 expression in the siPKM2 group (41.89 ± 5.54%), compared to the siControl group (55.76 ± 7.08%) (*P* < 0.05). Similarly, we observed a statistical difference in the expression of CD80 between the siControl group (43.79 ± 9.67%) and siPKM2 group (31.92 ± 7.41%) (*P* < 0.05, Figure 4).

### 3.3. PKM2-Mediated mDC Hyperfunction Is Required to Activate CD8+ T Cells from SAA Patients

**Figure 5:**
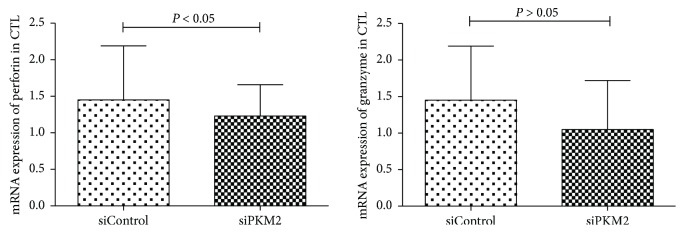
mRNA expressions of *perforin* and *granzyme B* in CTL cells by quantitative RT-PCR. The mRNA levels of *perforin* genes were identified significantly higher in the control group relative to the PKM2-siRNA group (*P* < 0.05). There was no statistical difference between the two groups in the expression level of *granzyme B* (*P* > 0.05).

The purity of sorted (see Materials and Methods) CD8+ T cells (CTLs) from SAA patients exceeded 90% in a flow cytometric analysis. To address the effect of PKM2 on the mDC-mediated activation of CTLs, mDCs from the PKM2-siRNA and siControl groups were cocultured individually with CTL cells, and the expression of *perforin* and *granzyme B* mRNA in CTL cells was analyzed by quantitative RT-PCR. As shown in Figure 5, the level of *perforin* mRNA was significantly higher in the siControl group (1.23 ± 0.43) relative to the PKM2-siRNA group (0.84 ± 0.39) (*P* < 0.05). The level of *granzyme B* mRNA was also higher (1.45 ± 0.74) in CTLs exposed to the siControl group relative to the PKM2-siRNA group (1.05 ± 0.67), although this difference was not significant (*P* > 0.05).

**Figure 6:**
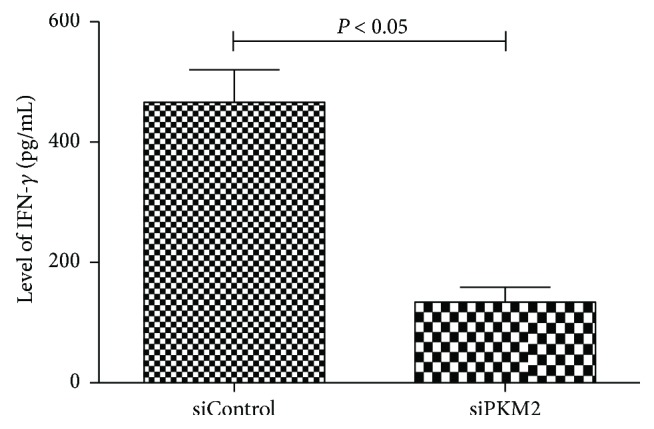
ELISA kits for quantification of IFN-*γ* level in the coculture supernatant. The level of IFN-*γ* in the control group was higher than that in the PKM2-siRNA group (*P* < 0.05).

Next, we used commercial ELISA kits to quantify the levels of IFN-*γ* in the coculture supernatants. Notably, a significantly higher IFN-*γ* concentration was detected in supernatants from the control group (466.3 ± 53.9 pg/mL), compared to the PKM2-siRNA group (134.2 ± 25.1 pg/mL) (Figure 6, *P* < 0.05).

**Figure 7:**
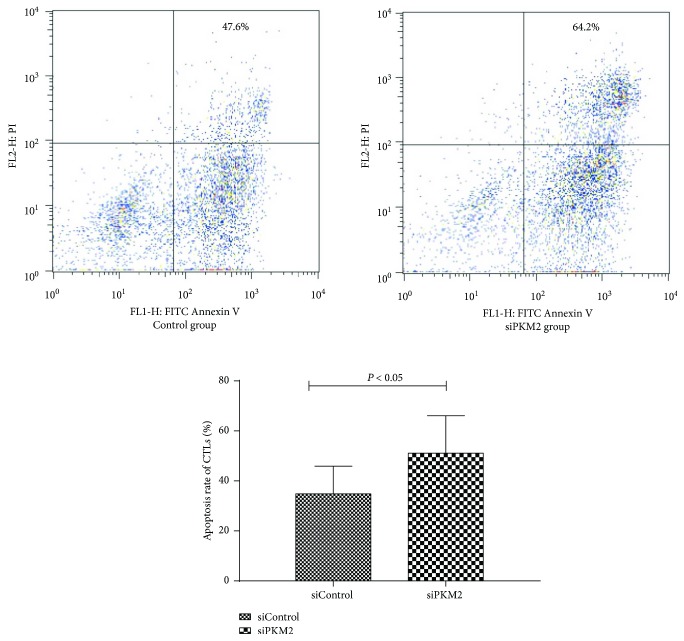
The apoptosis rate between the control group and siPKM2 group by flow cytometry using FITC Annexin V apoptosis detection kit I. Results showed that high PKM2 expression was significantly associated with reduced apoptosis rate (*P* < 0.05).

To evaluate the apoptosis of CTLs activated by mDCs after PKM2-siRNA transfection, we analyzed FITC Annexin V and propidium iodide (PI) staining via flow cytometry. Notably, high *PKM2* expression in mDCs correlated significantly with reduced CTL apoptosis. The mDCs induced CTL apoptosis in a manner consistent with the intrinsic level of PKM2 (Figure 7).

## 4. Discussion

SAA is characterized by severe pancytopenia and a bone marrow hematopoietic failure, with clinical symptoms including fatal anemia, infection, and bleeding. In recent years, the pathogenesis of SAA has been attributed to the dysregulation of immune cell subsets, particularly T lymphocytes, and the subsequently excessive apoptosis of hematopoietic stem cells [12]. Numerous studies have confirmed an abnormal cellular immune status in SAA patients, which includes the overactivation of CTLs, an imbalance of Th1/Th2 subsets resulting from the enhancement of Th1 and type I lymphoid factors, and insufficiencies in the regulatory T cell and NK cell population. Our previous study also found that the mDC populations in SAA patients were increased and overactivated [3]. However, the mDCs gradually returned to a normal state once disease recovery was achieved after IST [3]. As IST allows many SAA patients to achieve a good curative effect [13], we speculated that mDCs, as the most powerful professional antigen-presenting cells, might be abnormally stimulated during the primary stages of the immune response. In this study, we further subjected mDCs from SAA patients and normal controls to protein expression analyses and observed enhanced expression of PKM2 in cells isolated from SAA patients at an early stage after onset.

We used both western blotting and qPCR technology to detect PKM2 protein and mRNA expression in mDCs. Notably, we observed significantly higher levels of PKM2 expression at both the transcription and translation levels in mDCs from SAA patients, compared to normal controls, which preliminarily validated the results of our previous two-dimensional electrophoretic analysis. According to recent research, PKM2 exerts its strong immunomodulatory effect by interacting with suppressor of cytokine signaling 3 (SOCS3), thus disrupting the antigen-presenting abilities of dendritic cells [11]. Immune cells rely on a continuous supply of glucose to ensure the maintenance of normal functions. While interacting with antigen-presenting cells, T cells experience not only an increased glucose uptake rate but also an accelerated glycolytic metabolism rate [14]. Anomalies in glycolysis resulting from a pyruvate kinase deficiency limit the metabolic plasticities of specific immune cells and interfere with the abilities of these cells to eliminate intracellular pathogens [15, 16]. As noted in the Introduction, previous observations have suggested an important role for the nuclear translocation of PKM2 and the interaction of this protein with some pathogen-related proteins at the chromatin level in the context of disease progression [8–10]. Increasing evidence also suggests that PKM2 may serve as a critical regulator of immune cell metabolism by increasing or decreasing the Warburg effect, thus supporting a potential role for this protein in the genesis of inflammatory and autoimmune diseases [17].

Given the above results, we further assessed the effect of PKM2 on the functions of mDCs from patients with SAA. The mDC exerts its characteristic functions of antigen uptake and processing through a large number of dendritic protrusions on the surface and can perform liquid-phase endocytosis to present extremely low concentrations of antigen [18]. In this study, we used siRNA technology to investigate the effects of targeted *PKM2* knockdown on mDCs from SAA patients. In vitro, siRNA-mediated *PKM2* knockdown significantly inhibited the functions of mDCs: after knockdown, the phagocytic capabilities of mDC cells were significantly weakened relative to the control group, and the dendrites on the knockdown mDCs were found to be significantly smaller and shorter via electron microscopy. An immunofluorescence microsphere phagocytosis assay, which indirectly reflects the antigen uptake ability, indicated that the antigen uptake and presentation abilities of mDCs are tuned in a manner consistent with the level of intrinsic PKM2.

mDCs sense invading pathogens through various pattern-recognition receptors (PRRs) [19]. Immature mDCs convert to mature mDCs after capturing, processing, and presenting both exogenous and endogenous antigens and subsequently express high levels of the costimulatory molecules CD80 and CD86. The activation of resting T cells and the consequent protective immune responses against specific antigens require costimulatory molecules such as CD80 and CD86 [20]. Studies of systemic lupus erythematosus and other autoimmune diseases have demonstrated significantly elevated expression levels of these costimulatory surface molecules [21]. Accordingly, we used flow cytometry to evaluate the expression of CD80 and CD86 on mDCs from SAA patients. We observed reduced levels of CD86 and CD80 expression in the PKM2-transfection group, as well as a negative correlation between the proportion of CD86/CD80 and the level of PKM2 expressed in mDCs. These findings suggest a downregulation of the antigen-presenting capacities of dendritic cells and, consequently, an effect on downstream effector T cell function.

To verify the effects of PKM2 on the ability of mDCs from SAA patients to activate CTLs, we cocultured mDCs from the PKM2-siRNA group or siControl group with CTL cells and observed significantly reduced levels of *perforin* and *granzyme B* mRNA in CTL cells exposed to *PKM2*-knockout mDCs. Furthermore, CTLs exposed to the control mDCs produced significantly larger amounts of IFN-*γ*, as determined from an ELISA of supernatants. Perforin and granzyme B, which are released from CTLs and NK cells, are considered responsible for target cell cytotoxicity. Perforin, a calcium-dependent pore-forming protein, is released in the presence of Ca^2+^ and aggregates to form pores on target cell membranes. Granzyme B enters the target cell through these pores and cleaves specific substrates to initiate DNA fragmentation and apoptosis [22]. In multiple studies of autoimmune diseases, such as multiple sclerosis, CTLs were found to express increased levels of perforin and granzyme B [23]. Therefore, our results suggest that PKM2 knockdown in mDCs weaken the abilities of these cells to activate CTLs, thus decreasing the secretion of cytotoxic factors.

Subsequent flow cytometric apoptosis assays also demonstrated a significant association of high *PKM2* expression in mDCs with reduced CTL apoptosis. In the immune system, activated T cells are eliminated by apoptosis, which plays an important role in maintaining peripheral T cell homeostasis and regulating the intensity and duration of an immune response. Therefore, apoptosis is an important mechanism by which the body induces immune tolerance to exogenous antigens or autoantigens. Accordingly, the reduced CTL apoptosis observed in SAA patients may reflect an enhanced CTL activation level, which would aggravate the immune imbalance. PKM2 might therefore improve the immune status of an SAA patient by supporting the functions of mDCs.

In conclusion, the pathogenesis of SAA involves abnormalities of the immune system. Our preliminary study observed increased PKM2 expression in the mDCs of SAA patients, and this may be an important contributor to the overactivation of mDCs and downstream CD8+ T cells in this population. In the future, we aim to confirm the specific mechanism by which PKM2 affects the functions of mDCs and thus alters the immune processes in SAA.

## Figures and Tables

**Table 1:** Primers used for quantitative real-time PCR detection.

Target gene	Primer sequences	Annealing temperature (°C)
PKM2	F:5′-GACCTGAATGCCAGCGTATC-3′ F:5′-ACCTACACCTCCAAGCCATC-3′	58

*β*-Actin	F:5′-TTGCCGACAGGATGCAGAA-3′ R:5′-GCCGATCCACACGGAGTACT-3′	56

**Table 2:** Primers used for quantitative real-time PCR detection.

Target gene	Primer sequences	Product (bp)
Perforin	F:5′GAGGAGAAGAAGAAGAAGCACAA-3′ R:5′-AGGGGTTCCAGGGTGTAGTC-3′	200

Granzyme B	F:5′-CCAGCAGTTTATCCCTGTGAA-3′ R:5'-CACCTCTTGTAGTGTGTGTGAGTG-3′	235

*β*-actin	F:5′-TTGCCGACAGGATGCAGAA-3′ R:5′-GCCGATCCACACGGAGTACT-3′	100
